# Magnetization reversals in core–shell sphere clusters: finite-element micromagnetic simulation and machine learning analysis

**DOI:** 10.1038/s41598-023-42498-z

**Published:** 2023-09-14

**Authors:** Hyeon-Kyu Park, Sang-Koog Kim

**Affiliations:** National Creative Research Initiative Center for Spin Dynamics and Spin‐Wave Devices, Nanospinics Laboratory, Research Institute of Advanced Materials, Department of Materials Science and Engineering, Seoul National University, Seoul, 151‐744 South Korea

**Keywords:** Magnetic properties and materials, Ferromagnetism, Ferromagnetism, Magnetic properties and materials, Computational methods

## Abstract

Recently developed permanent magnets, featuring specially engineered microstructures of inhomogeneous magnetic phases, are being considered as cost-effective alternatives to homogeneous single-main-phase hard magnets composed of Nd_2_Fe_14_B, without compromising performance. In this study, we conducted a comprehensive examination of a core–shell sphere cluster model of Ce-substituted inhomogeneous Nd_2-*δ*_Ce_*δ*_Fe_14_B phases versus homogeneous magnetic phases, utilizing finite-element micromagnetic simulation and machine learning methods. This involved a meticulous, sphere-by-sphere analysis of individual demagnetization curves calculated from the cluster model. The grain-by-grain analyses unveiled that these individual demagnetization curves can elucidate the overall magnetization reversal in terms of the nucleation and coercive fields for each sphere. Furthermore, it was observed that Nd-rich spheres exhibited much broader ranges of nucleation and coercive field distributions, while Nd-lean spheres showed relatively narrower ranges. To identify the key parameter responsible for the notable differences in the nucleation fields, we constructed a machine learning regression model. The model utilized numerous hyperparameter sets, optimized through the very fast simulated annealing algorithm, to ensure reliable training. Using the kernel SHapley Additive eXplanation (SHAP) technique, we inferred that stray fields among the 11 parameters were closely related to coercivity. We further substantiated the machine learning models’ inference by establishing an analytical model based on the eigenvalue problem in classical micromagnetic theory. Our grain-by-grain interpretation can guide the optimal design of granular hard magnets from Nd_2_Fe_14_B and other abundant rare earth transition elements, focusing on extraordinary performance through the careful adjustment of microstructures and elemental compositions.

## Introduction

Demagnetization curves, magnetization hysteresis curves on the second quadrant, provide useful information on the characteristic properties of hard-magnetic materials^1–3^. The magnetic energy product *BH* and its maximum (*BH*)_max_, or magnetostatic energy can be calculated from the demagnetization curves, besides the remanence and coercivity that can be directly obtained from them. In this respect, the demagnetization curves of granular hard magnets are the key to understanding magnetization reversals and enhancing the performance of hard magnets. Thus, the characteristic shape of demagnetization curves has been intensively studied from earlier theoretical works including macrospin approximation^4,5^, Sharrock equation^6–8^, linear response theory^9^, Avrami kinetics^10^, Jiles-Atherton equation^2,11^, Preisach formalism^12^, and micromagnetic theory^1,13,14^. The micromagnetic theory and simulations allow determining demagnetization curves (or hysteresis curves) according to a variety of factors including intrinsic material parameters^15,16^, microstructures^12,17–31^, and damping constant and field sweep rate^8,26^, thus helping understand correlations between the demagnetization curve and the microstructures/compositions of granular magnets. For example, the microstructure effects of individual grains on the macroscopic performance of hard magnets have been extensively investigated in terms of the size^20,23–25^, crystallographic orientation of grains^18–24^, as well as precipitated phases within grain boundaries, and their thickness^15,24,27–31^. Very recently, several machine-learning models have also revealed the correlations between coercivity and (*BH*)_max_, switching field, and hysteresis loop with microstructure fingerprints. This AI-driven understanding of key microstructure factors has played a pivotal role in custom designs of the internal microstructures of hard magnets^15,16,20,24^.

In this context, it is intriguing to explore the underlying physics of magnetization reversals in core–shell multi-main phase (MMP) magnets. Such magnets were fabricated from conventional magnetic materials during dual-alloy processes, with the aim of substituting the costly neodymium (Nd) element with more abundant lanthanides such as La and Ce^30,32,33^. For example, it was reported that the MMP magnets incorporating 36 wt% La-Ce exhibited ~ 27% higher cost performance than that of the single-main-phase magnets with the same composition, albeit with a roughly 15% reduction in the (*BH*)_max_ value^32^. The enhanced cost performance was attributed to exchange coupling at the interfaces and intergranular dipolar interaction, according to measurements of the Curie temperature and recoil curves.

Also, several machine learning models were applied in studies of hard-magnetic materials to deduce the input-to-output relationships of magnetic properties, even with a relatively small number of datasets^15,16,20,22,24^. However, these models often have the limitation of not explicitly revealing the internal parameters, especially in the case of highly accurate models such as neural networks. Explainable artificial intelligence (XAI) techniques offer a solution by providing a set of numbers known as importance values. These values quantify the contribution of each input feature to the model’s output. The importance values can be extracted using specific algorithms, including the kernel SHapley Additive exPlanations (SHAP)^34^.

In this study, we utilized a novel approach, a grain-by-grain analysis of the demagnetization curves of all individual grains in given MMP magnets, in order to understand the underlying reversal mechanism of MMP magnets contributing to the overall demagnetization curve of the entire volume. We applied this grain-by-grain analysis to datasets obtained from finite-element micromagnetic simulations. These simulations were conducted on a core–shell sphere cluster model with various inhomogeneous magnetic phases of Nd_2_Fe_14_B and NdCeFe_14_B, each with different shell compositions surrounding each core sphere. Further, we discovered that the overall nucleation fields and coercivity were divided into two distinct broad and narrow distributions for the Nd-rich and Nd-lean individual grains, respectively. We attributed these differences to the stray fields resulting from the dipolar interactions of the individual spheres, which influence the nucleation field of the reversed domain in each sphere.

According to the kernel SHAP analysis of machine-learning models constructed for the coercivity of either Nd-rich or -lean grains, the magnitude of stray fields and the position of grains were the major factors contributing to the broader distributions of coercivity in Nd-rich grains. Such AI model interpretations align with the findings that adjustments to the shell compositions of both grain types can manipulate nucleation and coercive fields via intergranular magnetostatic interactions. The role of magnetostatic interactions was further explained by an analytical nucleation model composed of two hard-magnetic spheres.

## Results and discussion

### Core–shell sphere cluster model with inhomogeneous magnetic phases

**Figure 1:**
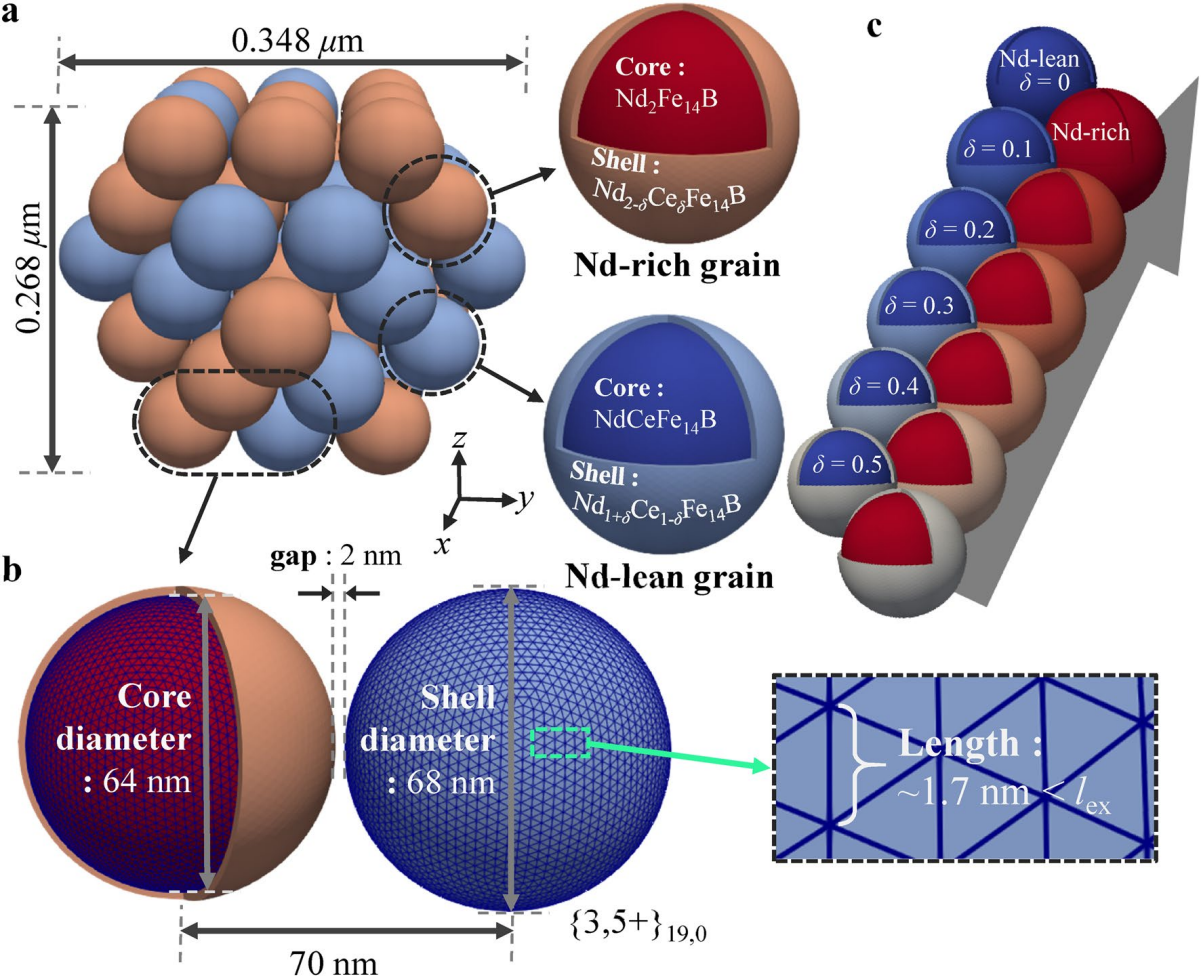
(**a**) Perspective view of a sphere-cluster model, each sphere featuring a core–shell structure along with the indicated dimensions. (**b**) The dimensions of the core–shell structure within each sphere. Individual spheres are separated by a 2-nm thick nonmagnetic medium. The inset shows the surface meshes of each sphere in the Class-I geodesic polyhedron, {3,5 +}_19,0_. (**c**) A cuboctahedron cluster model consisting of 28 Nd-rich spheres (red-tinted color for Nd_2_Fe_14_B) and 27 Nd-lean spheres (blue color for NdCeFe_14_B), each covered by a thin shell of Nd_2-*δ*_Ce_*δ*_Fe_14_B (pink) and Nd_1+*δ*_Ce_1-*δ*_Fe_14_B (light blue), respectively.

Our model studied here is composed of 55 spherical grains arranged in a double-layered cuboctahedron configuration with a core–shell structure^30,32^ in each sphere (Fig. 1a). Among various truncated octahedrons, the cuboctahedron possesses a sphericity of 0.905, close to 1. The cuboctahedron cluster comprises a specific number of spheres given by (2*n* + 1)(5*n*^2^ + 5*n* + 3)/3, where *n* (= 0, 1, 2,..) is the number of layers in the cluster model^35^. The sphere cluster model was designed to have a demagnetization factor of 1/3 in all directions^36–38^, thus eliminating possible shape anisotropy from the overall cluster volume. Each spherical grain has a 68 nm diameter with a 2-nm-thick shell, and each sphere was separated from its neighboring grains by a 2-nm air gap (Fig. 1b). We note that our model did not incorporate the soft or nonmagnetic defects that could serve as nucleation sites and pin domain walls, thereby potentially leading to an overestimated coercivity compared to experimental values^39,40^. The 68 nm diameter is considerably larger than the critical diameter (19.7 nm for Nd_2_Fe_14_B) for coherent magnetization rotation, but is smaller than a diameter (201 nm for Nd_2_Fe_14_B) above which multi-domain states are prevalent^14^. The air gap between neighboring grains inhibits short-range exchange coupling between them, thereby behaving as a nonmagnetic phase^28–30^.

To emulate the inhomogeneous phases of MMP magnets, our sphere cluster model consisted of two cores with distinct compositions—Nd_2_Fe_14_B (Nd-rich) and NdCeFe_14_B (Nd-lean), each enveloped by a single shell of Nd_2-*δ*_Ce_*δ*_Fe_14_B and Nd_1+*δ*_Ce_1-*δ*_Fe_14_B (0 ≤ *δ* ≤ 0.5), respectively. Notably, 28 Nd-rich spheres and 27 Nd-lean spheres were randomly dispersed as illustrated in Fig. 1c. In this model, we assume that the net content of Ce in the two different shells encompassing the Nd-rich and -lean cores are conserved, irrespective of given values of *δ*. The configurations of the Nd-rich and Nd-lean spheres were kept constant while *δ* was varied within a range of 0–0.5 at increments of 0.1. We assumed that our core–shell microstructures were formed by diffusion processes between Nd_2_Fe_14_B and NdCeFe_14_B particles mixed at 5:5 ratio, with the same diffusivity for Nd and Ce atoms. In this scenario, Nd atoms from the Nd_2_Fe_14_B particle and Ce atoms from the NdCeFe_14_B particle were presumed to exchange at a 1:1 rate. As such, the possible compositions of the Nd-rich and Nd-lean shell are anticipated to be Nd_2-*δ*_Ce_*δ*_Fe_14_B and Nd_1+*δ*_Ce_1-*δ*_Fe_14_B (0 ≤ *δ* ≤ 0.5), respectively.

### Demagnetization curves

**Figure 2:**
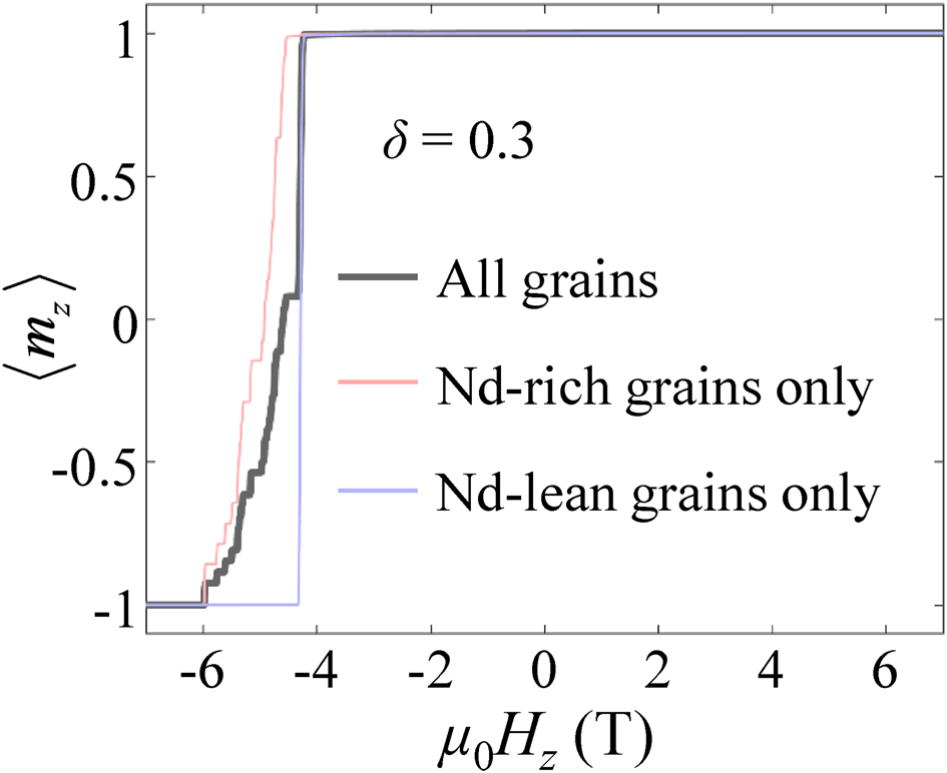
Demagnetization curves obtained from the core–shell sphere-cluster model with *δ* = 0.3. The pink-, light-blue-, black lines correspond to the normalized curves of Nd-rich spheres, Nd-lean spheres, and all spheres, respectively.

Figure 2 shows an example of simulation results for the overall demagnetization curve (thick black line) of all the spheres (entire cluster model system), and two separate demagnetization curves exclusively representing the Nd-rich and -lean spheres for the case of *δ* = 0.3. The overall demagnetization curve exhibits a significant, sudden drop in < *m*_*z*_ > just beyond the nucleation field *H*_*N*_, succeeded by a series of relatively smaller-step curves. This type of curve is typical for a reversal process of the nucleation of reversed domains, as is often observed in exchange-decoupled magnets^28,29^. The entire demagnetization curve can be dissected into two separate curves, obtained from only the Nd-rich and -lean spheres, depicted in red and blue colors, respectively. Notably, these two decomposed demagnetization curves exhibit stark contrasts: a single pronounced step-drop for the Nd-lean spheres versus numerous minor step-drops for the Nd-rich spheres. In detail, the Nd-lean spheres show a sharp, significant drop in the magnetization at* μ*_0_*H*_z_ =  ~ − 4.3 T, while the Nd-rich spheres shows a few moderate-step drops before *H*_*c*_ but many minor step drops after *H*_*c*_. Therefore, the entire-system value of *H*_*c*_ is the cumulative result of the reversals of the Nd-rich and -lean spheres and is dominated by the switching of the Nd-rich spheres. Moreover, the reversal process of the Nd-rich spheres was composed of a sequence of successive switching of the individual Nd-rich spheres, each with different nucleation fields, across a wide range of *H*_*N*_. The sequential reversal processes of Nd-lean and -rich grains are visually depicted in Supplemental Movie S1, available online.

### Grain-by-grain analysis of demagnetization curves and the reversals of individual spheres

To understand the overall demagnetization curve characterized by numerous step-like drops in magnetization shown in Fig. 2, we performed a grain-by-grain analysis, separating the demagnetization curves of individual spheres. Because the multiple steps observed in the overall demagnetization curve result from different nucleation fields required for switching reversed domains in each individual sphere, we interpreted the demagnetization curves sphere-by-sphere.

**Figure 3:**
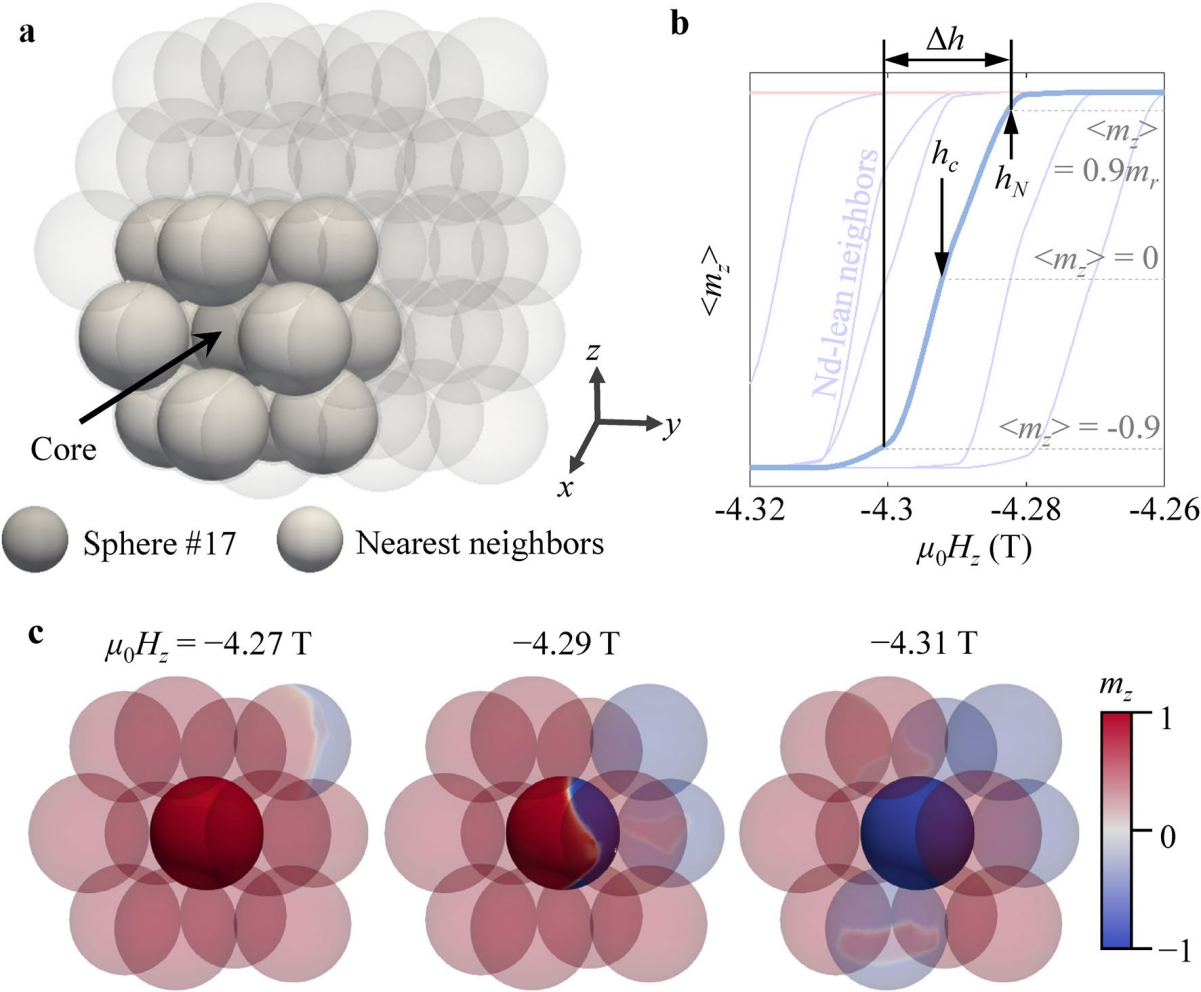
Representation of grain-by-grain analysis for demagnetization curves. (**a**) Highlight of 12 spheres surrounding a single sphere, labeled as #17. (**b**) Demagnetization curves of several individual spheres, along with the coercive force *h*_*c*_, the nucleation field *h*_*N*_, and its slope Δ*h* as defined within the diagram. The thick blue line indicates the demagnetization curve of sphere #17, while thin lines correspond to those of its neighboring spheres. The snapshot images in (**c**) describe the temporal magnetizations at the indicated values of external magnetic fields. The colors indicate *m*_*z*_ as indicated by the color bar.

Figure 3a highlights parts of the cluster model, emphasizing a Nd-lean sphere labeled as #17 and its twelve nearest neighboring (NN) spheres. The demagnetization curves for sphere #17 and some of the NN spheres are separately plotted in Fig. 3b. The *μ*_0_*H*_*c*_ values for sphere #17 and five Nd-lean spheres ranged from 4.26 to 4.32 T. Figure 3c shows snapshot images of local *z*-component magnetizations (*m*_*z*_) distributions at *μ*_0_*H*_z_ = − 4.27, − 4.29, and − 4.31 T for sphere #17 and the twelve NN spheres. The magnetization reversal occurred sphere-by-sphere via the individual switching of each sphere, similar to exchange-decoupled magnets^28,29^, although the reversal in sphere #17 was not coherent. To quantitatively interpret the switching of individual spheres, demagnetization curves are plotted to represent the varying values of coercive field *h*_c_, nucleation field *h*_*N*_, and field width Δ*h* as illustrated for different spheres. The parameters *h*_*c*_ and *h*_*N*_ were defined as the fields obtained at < *m*_*z*_ >  = 0 and < *m*_*z*_ >  = 0.9*m*_*r*_, respectively, while Δ*h* is defined as the difference in *μ*_0_*H*_*z*_ between < *m*_*z*_ >  = 0.9*m*_*r*_ and − 0.9. Since the reversal of each sphere in our model is incoherent within the volume of each sphere, the value of Δ*h* also approximately measures the mobility of domain walls within each sphere.

### Distributions of *h*_*c*_, *h*_*N*_, and Δ*h* for all individual spheres

**Figure 4:**
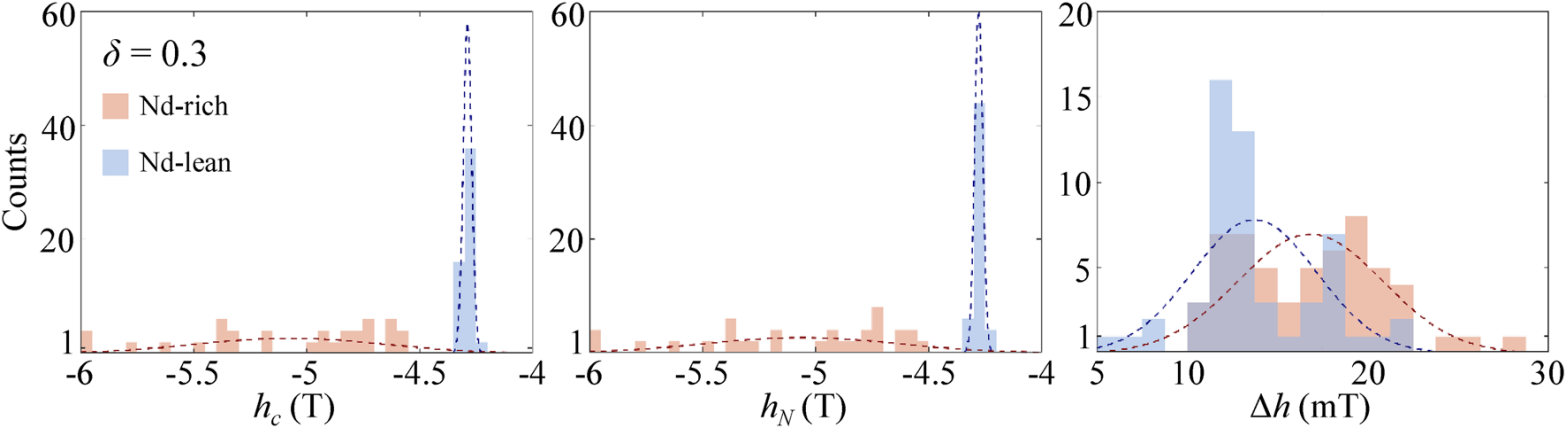
Histograms displaying the distributions of *h*_*c*_, *h*_*N*_, and Δ*h* values of all individual spheres in the case of *δ* = 0.3. The red bars indicate Nd-rich spheres, while the blue bars denote Nd-lean spheres. Dotted lines represent the fitted normal distributions of *h*_*c*_, *h*_*N*_, and Δ*h* for both Nd-rich and -lean spheres.

The distributions of all the *h*_c_, *h*_*N*_, and Δ*h* values for the Nd-rich (red color) and Nd-lean (blue) spheres in the core–shell sphere cluster model with an inhomogeneous composition of *δ* = 0.3 are depicted as histograms (Fig. 4). The normal distribution curves (*f*^*R*^ and *f*^*L*^) for the Nd-rich and -lean spheres are separately plotted, accompanied by the corresponding mean and standard deviation values calculated using the following equations.

1a $$f^{R} (h_{c} ) = \frac{{N_{R} b}}{{\sqrt {2\pi \sigma_{{h_{c} }}^{R} } }}\exp \left[ { - \frac{{\left( {h_{c} - \left\langle {h_{c} } \right\rangle^{R} } \right)^{2} }}{{2\left( {\sigma_{{h_{c} }}^{R} } \right)^{2} }}} \right]$$

1b $$f^{L} (h_{c} ) = \frac{{N_{L} b}}{{\sqrt {2\pi \sigma_{{h_{c} }}^{L} } }}\exp \left[ { - \frac{{\left( {h_{c} - \left\langle {h_{c} } \right\rangle^{L} } \right)^{2} }}{{2\left( {\sigma_{{h_{c} }}^{L} } \right)^{2} }}} \right]$$

where *N*_*R*_ (*N*_*L*_) represents the number of the Nd-rich (Nd-lean) spheres, including both core and shell (56 for Nd-rich and 54 for Nd-lean spheres in this model), *b* denotes the bin width of the histograms, while $\left\langle {h_{c} } \right\rangle^{R}$ ($\left\langle {h_{c} } \right\rangle^{L}$) and $\sigma_{{h_{c} }}^{R}$ ($\sigma_{{h_{c} }}^{L}$) refer to the mean value and the standard deviation of *h*_c_, respectively. The fitting values of the mean and standard deviations were summarized in Supplementary Table S1 online. Normal distribution curves of *h*_*N*_ and Δ*h* were obtained in the same manner. The *h*_*c*_ and *h*_*N*_ of the Nd-rich grains were distributed over a wide range from − 6 to − 4.5 T, with the mean values of $\left\langle {h_{c} } \right\rangle^{R} = - 5.07{\text{ T}}$ and $\left\langle {h_{N} } \right\rangle^{R} = - 5.06{\text{ T}}$, and standard deviations of $\sigma_{{h_{c} }}^{R} = 416.7{\text{ mT}}$ and $\sigma_{{h_{N} }}^{R} = 418.0{\text{ mT}}$, respectively. In contrast, the Nd-lean spheres displayed relatively narrow distributions centered around $\left\langle {h_{c} } \right\rangle^{L} = - 5.07{\text{ T}}$ and $\left\langle {h_{N} } \right\rangle^{L} = - 5.06{\text{ T}}$, and standard deviations of $\sigma_{{h_{c} }}^{L} = 18.5{\text{ mT}}$ and $\sigma_{{h_{N} }}^{L} = 17.8{\text{ mT}}$, respectively. Hence, the many step-like demagnetization curve for the Nd-rich grains (as seen Fig. 2) is attributed to the variation in nucleation fields across a wide range. The extremely broad ranges of *h*_c_ and *h*_*N*_ for the Nd-rich grains will be explained in the following section, with reference to the uneven distribution of stray fields affecting each Nd-rich grain^14^. On the other hand, the similar and narrow distributions of $\left\langle {\Delta h} \right\rangle^{R} = 19.5{\text{ mT}}$ and $\left\langle {\Delta h} \right\rangle^{L} = 17.0{\text{ mT}}$ with $\sigma_{\Delta h}^{R} = 3.20{\text{ mT}}$ and $\sigma_{\Delta h}^{L} = 2.76{\text{ mT}}$ for both the Nd-rich and -lean grains indicate comparable domain wall mobilities in both types of grains. According to the one-dimensional model, the speed of domain walls is expressed as

2 $$v_{DW} = \frac{{\gamma \mu_{0} \alpha \delta_{DW} }}{{1 + \alpha^{2} }}H_{ext}$$

where *δ*_*DW*_ is the domain wall width and *H*_*ext*_ the applied magnetic field driving the domain walls^46^. The width of domain wall in a curved geometry depends on the curvature value, the position within each sphere particle, as well as the direction of domain wall expansion relative to the crystallographic orientations^1,18,42–44^. Taking into account the Bloch domain wall width $\delta_{DW} = \pi \sqrt {{{A_{ex} } \mathord{\left/ {\vphantom {{A_{ex} } {K_{1} }}} \right. \kern-0pt} {K_{1} }}}$ for core regions and the mean coercive fields as the driving field values (*δ*_*DW*_ = 1.34 and 1.48 nm; |< *h*_*c*_ >|= ~ 5.07 and ~ 4.29 T for Nd-rich and Nd-lean grains, respectively), the values of *v*_*DW*_ for the Nd-rich and -lean grains are estimated to be 1.88 km/s and 1.75 km/s, respectively. The *v*_*DW*_ values differ only by + 7.0% ($v_{DW}^{R} > v_{DW}^{L}$) between the two types of grains. This is a compensated result of − 10.6% and + 18.3% differences in the values of *δ*_*DW*_ ($\delta_{DW}^{R} < \delta_{DW}^{L}$) and driving fields ($\left\langle {h_{c} } \right\rangle^{R} > \left\langle {h_{c} } \right\rangle^{L}$).

### Explaining the broader distribution of *h*_*c*_ in Nd-rich grains by machine learning approach

To identify the mechanism behind the broad distributions of *h*_*N*_ and *h*_*c*_ observed in Nd-rich grains, we constructed machine learning models based on artificial neural networks. We then extracted the feature importance values, which quantitatively measure the influence of features on the model’s output, using kernel SHAP interpretation^34^. Previous studies^20^ have identified crystallographic misorientations and relative position of grains as key features determining each grain’s switching field. In a similar manner, we compiled 11 features characterizing each grain, which include the material parameters (*x*_Ce_), the relative position of grains (*r*_*x*_, *r*_*y*_, *r*_*z*_, *r*), the number of neighboring grains of different types (*NN*_*rich*_, *NN*_*lean*_), and the mean stray field acting on each grain ($H_{stray}^{x}$, $H_{stray}^{y}$, $H_{stray}^{z}$, $H_{stray} = \sqrt {(H_{stray}^{x} )^{2} + (H_{stray}^{y} )^{2} + (H_{stray}^{z} )^{2} }$). The vector stray field acting on the *i*-th grain was calculated using an approximate macrospin model that employs volume-average *z*-component magnetizations^45^: ${\mathbf{H}}_{{{\text{stray}}}}^{i} = - \sum\limits_{j \ne i} {\nabla \Phi_{M}^{ij} }$ with the magnetic scalar potential

3 $$\Phi_{M}^{ij} = \frac{{J_{S}^{{j,{\text{core}}}} \left\langle {m_{z}^{{j,{\text{core}}}} } \right\rangle }}{{3\mu_{0} }}\frac{{R_{{{\text{core}}}}^{3} }}{{r_{ij}^{2} }}\cos \theta_{ij} + \frac{{J_{S}^{{j,{\text{shell}}}} \left\langle {m_{z}^{{j,{\text{shell}}}} } \right\rangle }}{{3\mu_{0} }}\frac{{R_{{{\text{shell}}}}^{3} - R_{{{\text{core}}}}^{3} }}{{r_{ij}^{2} }}\cos \theta_{ij} ,$$

where $J_{S}^{{j,{\text{core}}}}$ ($J_{S}^{{j,{\text{shell}}}}$), $\left\langle {m_{z}^{{j,{\text{core}}}} } \right\rangle$ ($\left\langle {m_{z}^{{j,{\text{shell}}}} } \right\rangle$), and $R_{{{\text{core}}}}$ ($R_{{{\text{shell}}}}$) are the saturation polarization, volume-average *z*-component magnetization, radius of core (shell) part of the *j*-th grain, and $r_{ij}$ and $\theta_{ij}$ are the center-to-center distance and angle between the *i*- and *j*-th grains. The stray fields closely corresponded with the demagnetizing fields calculated from the micromagnetic simulations, as exemplified in the case of sphere #17, shown in Supplementary Fig. S1 online.

**Figure 5:**
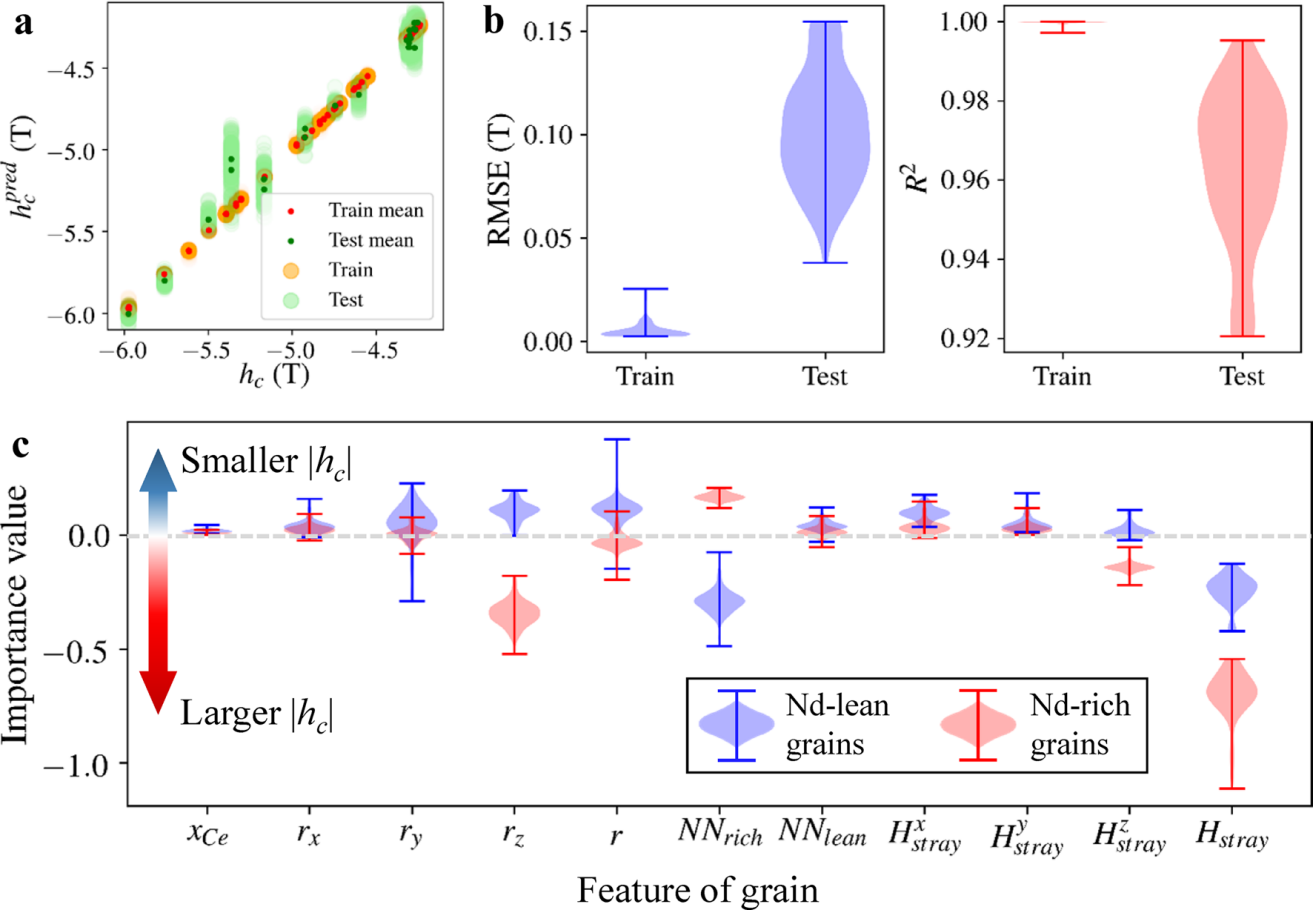
Machine learning approach for analyzing coercivity variation. (**a**) Parity plot comparing the original simulation data (*h*_*c*_) with the predictions made by 100 machine learning models ($h_{c}^{pred}$) using different sets of hyperparameters optimized by VFSA. The large dots shaded in orange and green indicate the predictions for the train and test datasets, respectively, while the small dots in red and deep-green represent the mean of predictions for the train and test datasets, respectively. (**b**) Violin plots depicting the RMSE and *R*^2^ between the simulation datasets and predictions for the train and test datasets made by the 100 models. (**c**) Kernel SHAP interpretation reveals the importance values of the 11 features used to train the models. Features with negative importance values enhance the |*h*_*c*_|, while those with positive values reduce |*h*_*c*_|.

Using the 11 features, we trained 100 artificial neural network models with different sets of hyperparameters, optimized by the very fast simulated annealing (VFSA) algorithm^24^. The optimized models accurately reproduced prediction values comparable to those of the original datasets (Fig. 5a). Predictions had a root mean square error (RMSE) of 5.3 (± 4.2) mT and 102.4 (± 26.9) mT, and an *R*^2^ score of 0.99 (± < 0.01) and 0.96 (± 0.019) for the training and test datasets, respectively (Fig. 5b). By employing the kernel SHAP interpretation method, we extracted the importance values of the 11 features, which are measures of their contributions to the model’s prediction^34^. Therefore, a negative importance value contributes to a larger |*h*_*c*_| and a positive one to a smaller |*h*_*c*_|.

In Fig. 5c, the importance values, calculated from the *h*_*c*_ prediction of the 100 neural network models, are summarized in violin plots with whiskers indicating maximum, medium and minimum values. Among the 11 features, the contribution by the stray field magnitude (*H*_*stray*_) was the most important factor for Nd-rich grains and the second most important for Nd-lean grains, with importance values of − 0.706 and -0.243, respectively. The number of neighboring Nd-rich grains (*NN*_*rich*_) was the key factor that most increased the |*h*_*c*_| of the Nd-lean grains, with the value of − 0.29, but it was also the factor that most decreased the |*h*_*c*_| of the Nd-rich grains, with the value of + 0.169. In contrast, the *z*-position of the grains (*r*_*z*_) decreased |*h*_*c*_| of Nd-lean grains (importance value: + 0.105) and increased |*h*_*c*_| of Nd-rich grains (− 0.344). However, the material nature of the grain itself (*x*_Ce_) had negligible effects on |*h*_*c*_| of either type of grains (+ 0.02 for Nd-lean, + 0.01 for Nd-rich grains).

The |*h*_*c*_| of Nd-rich grains was influenced more significantly by *H*_stray_ and *r*_*z*_ (importance values: − 0.706, − 0.344) than the |*h*_*c*_| of Nd-lean grains (− 0.243, + 0.105). Consequently, the broader distributions of *h*_*c*_ in Nd-rich grains can be attributed to the stray field and the *z*-position of each grain. As referenced in studies^20,24^, the latter was one of the most prevalent features of weak grains and resulted in anomalously small values of switching field and magnetic energy products. Though the importance values of *H*_*stray*_ and *r*_*z*_ had similar trends, they showed weak correlation (*ρ* = 0.06) in our model. In the latter part of this paper, we will analyze the switching field (or *h*_*c*_) from the perspective of stray fields (*H*_*z*_). This interpretation will be based on an eigenvalue problem rooted in micromagnetic theory.

### Grain-by-grain analysis for inhomogeneous magnetic phases

**Figure 6:**
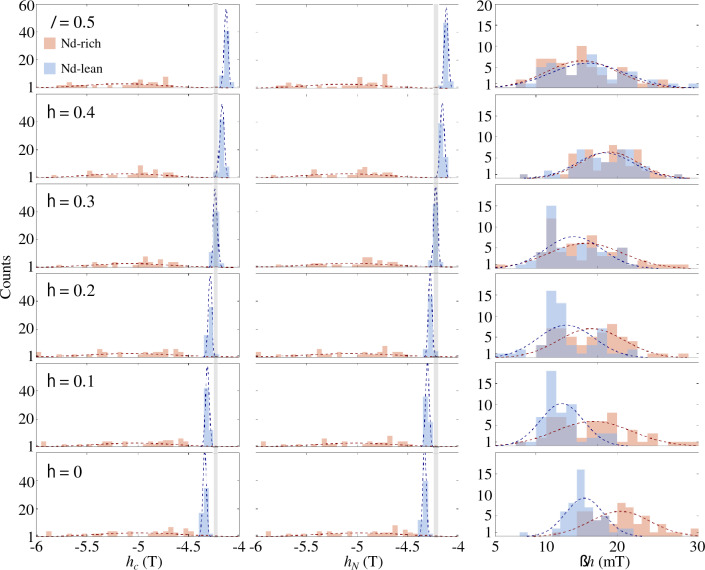
Histograms depicting the distributions of *h*_*c*_, *h*_*N*_, and Δ*h* values for individual spheres with different values of *δ* (= 0, 0.1, 0.2, 0.3, 0.4, and 0.5). The vertical gray lines in the *h*_*c*_ and *h*_*N*_ histograms indicate the corresponding median values for *δ* = 0.3.

**Figure 7:**
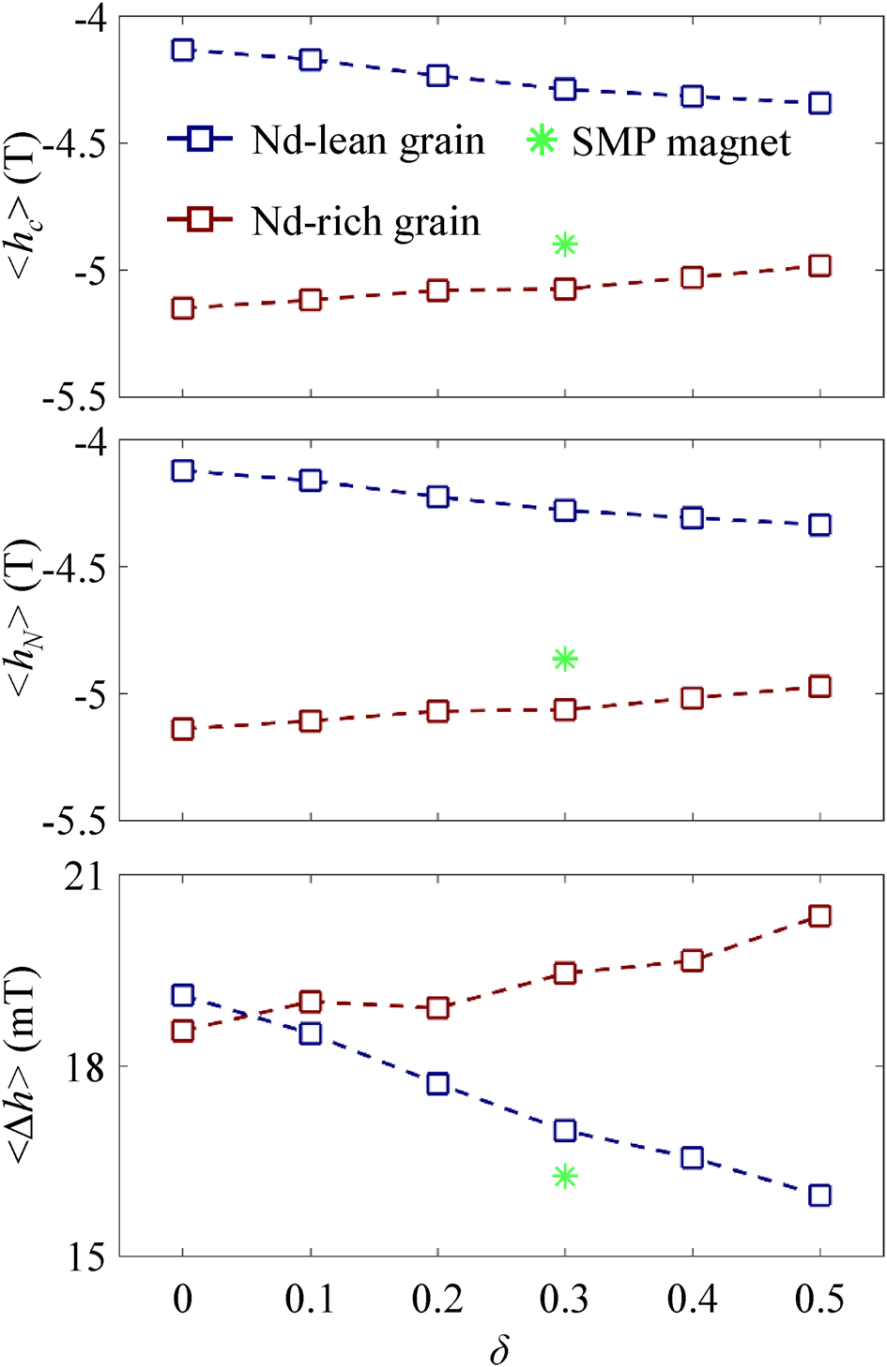
Plots of < *h*_*c*_ > , < *h*_*N*_ > , and < Δ*h* > averaged for the Nd-rich and Nd-lean spheres as a fucntion of *δ*. The green asterisk indicates those for the single-main-phase sphere cluster model of *δ* = 0.3.

**Figure 8:**
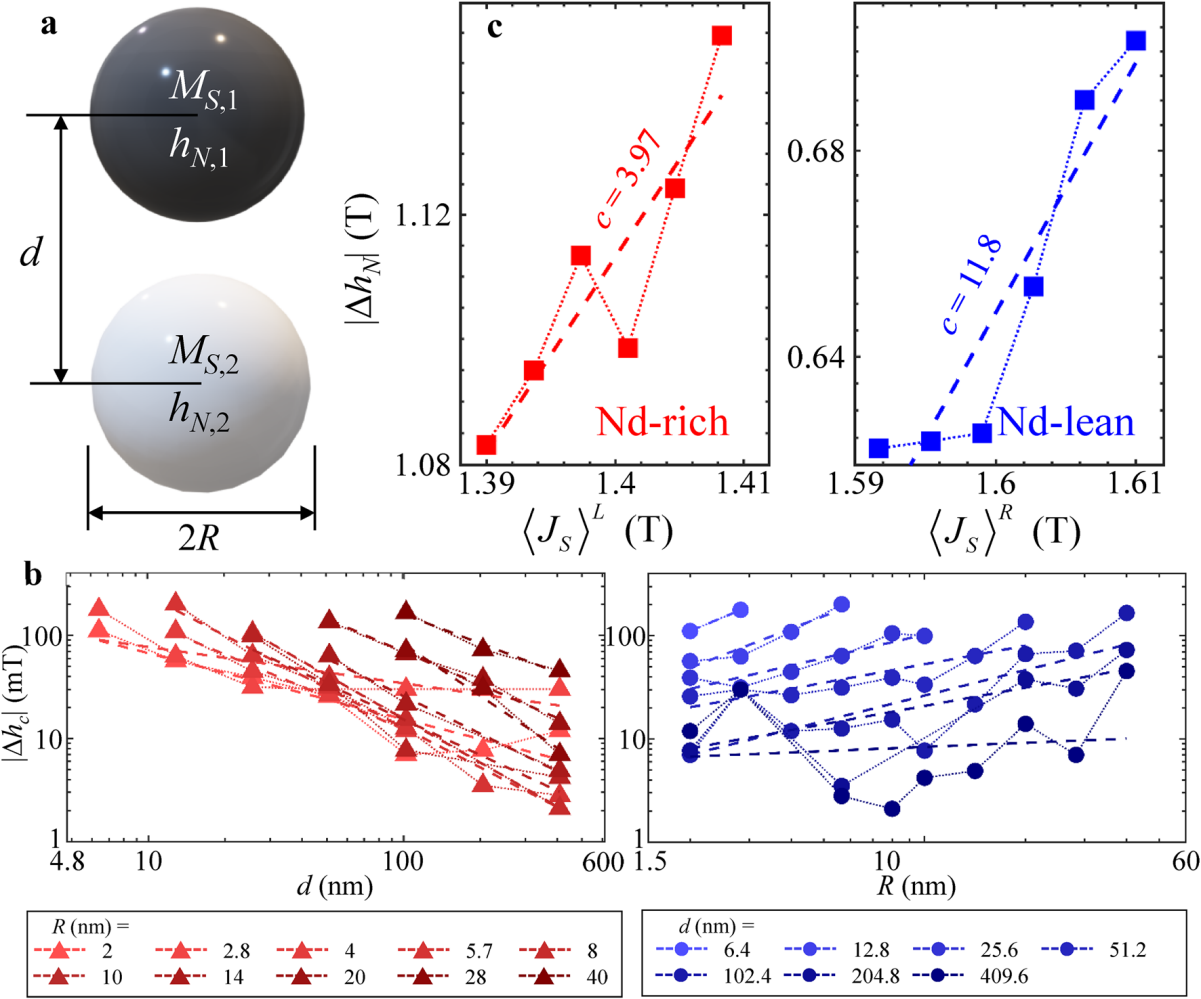
The coupled Stoner-Wohlfarth model. (**a**) The model geometry of vertically aligned magnetic particles of a radius *R*, separated by an interparticle gap of *d*. (**b**) The plot of the coercive field discrepancy in relation to the radius and interparticle gap, shown on logarithmic scales. Fitting curves using Eq. (6) are represented by dotted lines. (**c**) The plot of the nucleation field discrepancy in relation to the saturation polarization of surrounding grains, with fitting curves represented by dotted lines.

To account for the distinct dependencies of coercive forces and nucleation fields on *δ*, we examined the demagnetization curves using a grain-by-grain analysis, as shown in the *h*_c_, *h*_*N*_, and Δ*h* histograms (see Fig. 6). The mean and standard deviations of these parameters for different *δ* values are summarized in Supplementary Table S2 online and in Fig. 7, along with those from the single-main-phase model. As shown in Fig. 8, the means of *h*_c_ and* h*_*N*_ of Nd-lean grains ($\left\langle {h_{c} } \right\rangle^{L}$ and $\left\langle {h_{N} } \right\rangle^{L}$) decrease as *δ* values increase, aligned with the trend of overall nucleation fields. On the other hand, $\left\langle {h_{c} } \right\rangle^{R}$ and $\left\langle {h_{N} } \right\rangle^{R}$ increase with *δ*, following the same trend as the overall coercive forces. These variations can be explained in terms of an inverse dependence of anisotropy fields ($h_{A} = 2\mu_{0} K_{1} /J_{S}$) on *δ*, which monotonically varies from 6.71 (*δ* = 0) to 5.24 T (*δ* = 0.5), as indicated by the Kronmüller relation ($h_{c} = \alpha h_{A} - NJ_{S}$, *α* is the microstructure factor and *N* the effective demagnetization factor)^14^. The increase in Ce contents of shells (*X*_Ce_ = *δ*/2100 at%) suggests a depletion of Nd atoms from the shell of Nd-rich grains, leading to a decrease in anisotropy fields in the Nd-rich grains’ shell region, where reversed domains are initially nucleated. At the same time, the surplus Nd atoms are integrated into the shell region of Nd-lean grains, enhancing their anisotropy fields. We will discuss the *δ* dependence of $\left\langle {h_{N} } \right\rangle^{R(L)}$ in more detail in the next section, using empirical relations. To compare with parameters from the single-main-phase model, its $\left\langle {h_{c} } \right\rangle$ and $\left\langle {h_{N} } \right\rangle$ values are marked with a green asterisk, lying between the curves for Nd-rich and -lean grains due to the intermediate Ce content of the material assumed in our single-main-phase magnet model (Nd_1.5082_Ce_0.4918_Fe_14_B).

### Exploring the mechanism behind variations in *h*_*N*_ with *δ*

In the previous section, we found that the stray fields are the most essential among other features. However, relying solely on the results-driven machine learning model for this inference lacks a physics background. Therefore, in this section, we further established an analytical model that involves solving the nucleation problem in classical micromagnetic theory. It is worth noting that the nucleation field varies with different *δ*, although the overall chemical formula for the sphere cluster, Nd_2-*x*_Ce_*x*_Fe_14_B (0.491 ≤ *x* ≤ 0.5), has minimal differences with cerium stoichiometry varying at most by 1.8%. Apart from the chemical compositions, microstructural factors such as intergranular exchange stiffness, easy axis alignment, and grain sizes can cause discrepancies between experimental and ideal coercivity and/or nucleation fields, a problem well-known as Brown’s paradox^14,46,47^. In previous studies^14,48–50^, this discrepancy between experimental and ideal coercivity in granular materials was explained in relation to demagnetizing fields. The demagnetizing fields were linked to the physical characteristics of grains^20,49^ and the cavity field originating from the sheath of grains^48^ as demonstrated by Monte-Carlo simulations^50^. However, assessing coercivity through simulation methods has limitations, as the calculated coercivity depends on the dynamic features of the model system such as damping parameter and sweep rates^14^. Furthermore, these methods provide less detailed insights into the mechanism behind the relationship between the material parameters and coercivity than analytical expressions do.

Thus, we used an analytical macrospin model that accounts for inter-particle magnetostatic interaction energy to demonstrate that the stray field can reduce the coercivity of magnetically coupled particles. The nucleation field was calculated using the principle that the second derivative of the magnetic energy holds a zero eigenvalue when the nucleation of reversed domains begins^47,51^. We developed a model geometry composed of two hard-magnetic spheres with a radius *R* and a center-to-center distance *d*, both exhibiting uniaxial anisotropy (Fig. 8a). Around the point of nucleation, the magnetic energy of the two spheres, which are coupled through magnetostatic interactions, is approximately given as,

4 $$E(\theta_{1} ,\theta_{2} ) = \frac{{\mu_{0} M_{S,1}^{2} }}{2}(h - h_{N,1}^{ \circ } )\theta_{1}^{2} + \frac{{\mu_{0} M_{S,2}^{2} }}{2}(h - h_{N,2}^{ \circ } )\theta_{2}^{2} + \frac{2}{3}\mu_{0} M_{S,1} M_{S,2} \left( \frac{R}{d} \right)^{3} \cos \theta_{1} \cos \theta_{2}$$

where *θ*_*i*_, *M*_*S,i*_, and $h_{N,i}^{ \circ }$ (*i* = 1, 2) correspond to the spin angle, saturation magnetization, and ideal nucleation field of sphere *i*., respectively. The first and second terms are the Taylor expansions of Stoner-Wohlfarth particles up to the second order of *θ*_*i*_, on the verge of nucleation ($\theta_{i} \approx 0$), or

$$E_{SW} (\theta ) = K_{1} \sin^{2} \theta - \mu_{0} M_{S} h\cos \theta = \mu_{0} M_{S} \left( {\frac{{h_{A} }}{2}\sin^{2} \theta - h\cos \theta } \right) \approx E_{0} + \mu_{0} M_{S} (h - h_{N} )\frac{{\theta^{2} }}{2}$$

with $h_{N} = - h_{A} = - 2K_{1} /\mu_{0} M_{S} < 0$, representing the ideal nucleation field. Using the nucleation condition, which states that the determinant of $\tfrac{{\partial^{2} E}}{{\partial (\theta_{1} ,\theta_{2} )}}$ becomes zero at nucleation ($\theta_{1} ,\theta_{2} \approx 0$)*,* the nucleation fields of the *i*-th sphere in the coupled system ($h_{N,i}$) is given as

5 $$h_{N,1(2)} = h_{N,1(2)}^{ \circ } + \frac{2}{3}\left( \frac{R}{d} \right)^{3} M_{S,2(1)} .$$

Based on this dependency relation, the decrease in nucleation field in general systems with a grain radius *R*, interparticle distances *d*, and the saturation magnetization of the other particle as $\overline{M}_{S}$ should scale as follows:

6 $$\Delta h_{N} \sim - R^{a} d^{ - b} \overline{M}_{S}^{c} .$$

with positive exponents *a*, *b*, and *c*. To validate this scaling rule, we performed additional micromagnetic simulations. These simulations were conducted using the material parameters of Nd_2_Fe_14_B and NdCeFe_14_B, sphere radii (*R*) ranging from 2 to 40 nm, and center-to-center distance (*d*) from 6.4 to 409.6 nm, which satisfies $d > 2R$. The coercive fields were calculated by applying an external magnetic field in the -*z* direction to the spheres, which had initially been magnetized to ${\mathbf{m}} = [0,0,1]$. In Fig. 8b, the differences between the coercive fields of those systems and the coercive field of single spheres with the same dimensions are plotted, along with the fitting curves based on (6). The parameters *a* and *b* extracted from the datasets ranged from 0.13 to 1.35 and 0.35 to 1.68, respectively.

The empirical relation (6) was applied to the datasets in Fig. 8, aiming to illustrate the discrepancy between the nucleation fields calculated from simulations and the ideal nucleation fields as described in^47^. The values of $\left\langle {h_{N} } \right\rangle^{R(L)}$ for *δ* = 0, 0.1, 0.2, 0.3, 0.4, 0.5 were fitted with Eq. (6) in Fig. 8c. The discrepancies in the nucleation field of Nd-rich (-lean) grains ($\left| {\Delta h_{N} } \right|^{R(L)} = \left\langle {h_{N} } \right\rangle^{R(L)} - h_{N}^{ \circ }$) are plotted against the volume-average saturation magnetization of Nd-lean (-rich) grains, with both axes on a logarithmic scale. The volume-average saturation polarization, $\left\langle {J_{S} } \right\rangle_{V}^{L(R)}$, was calculated based on volume fractions, as follows:

$$\left\langle {J_{S} } \right\rangle_{V}^{L(R)} = v_{core} J_{S}^{core,L(R)} + v_{shell} J_{S}^{shell,L(R)} ,$$

With $v_{core}$, $J_{S}^{core,L(R)}$ and $v_{shell}$, $J_{S}^{shell,L(R)}$, representing the volume fraction and saturation polarization of Nd-rich (-lean) grains. The ideal nucleation field $h_{N}^{ \circ }$ for the curling rotation mode was calculated from $h_{A} = 2x_{1}^{2} \mu_{0} A_{ex} /J_{S} R^{2} + 2\mu_{0} K_{1} /J_{S} - J_{S} /3$ with $x_{1} = 2.0816$^50^. The *c* values, obtained by fitting Eq. (6) to $\left| {\Delta h_{N} } \right|^{R}$ and $\left| {\Delta h_{N} } \right|^{L}$ (Fig. 8c) were 3.97 and 11.8, respectively, both indicating a positive value of *c*.

According to our analytical nucleation model, the stray field alone can account for a large part of the discrepancy mismatch between the calculated and ideal coercivity values. By addressing an eigenvalue problem for the two-sphere model, we demonstrated that the introduction of a stray field, as a magnetostatic interaction term, results in decreases of nucleation fields. This finding is consistent with the interpretation given by the kernel SHAP of the machine-learning-based regression model shown in Fig. 5c. From a mathematical standpoint, the convex position of the energy function, which is approximately expanded by polynomials as shown in Eq. (4), is altered by the superposition of stray fields emanating from magnetic charges in other domains. Considering that the stray fields in uniformly magnetized spheres result from surface magnetic charges, the stray field model can explain the coercivity mechanisms of core–shell MMP magnets and potentially phenomena related to microstructure, such as Brown’s paradox.

Directly applying our macro-spin model to previously published experimental results on MMP magnets proved challenging. However, our model, which demonstrates a decrease in *h*_*N*_ with increase in *R* and a decrease in *d*, provides insights into previous experimental observations related to grain sizes and grain-to-grain exchange interactions. For example, the decrease in *h*_*c*_ with an increase in *R* has been attributed to surface defects, in analogue with structural mechanical weakest-link statistics^52,53^. On the other hand, the reduction in *h*_*c*_ with *d* (or increased inter-grain exchange coupling) was explained through micromagnetic simulations in earlier studies^24,28,29^.

### Comparison with the single-main-phase model

**Figure 9:**
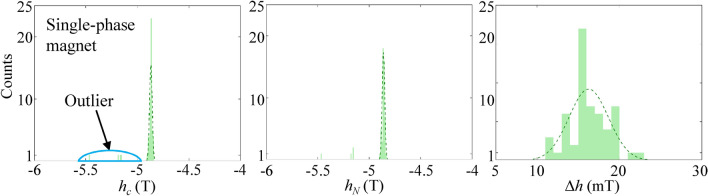
Histograms for the distributions of *h*_*c*_, *h*_*N*_, and Δ*h* values for all the individual spheres having a homogenous composition equivalent to the core–shell sphere cluster model of *δ* = 0.3. Dotted lines represent the normal distributions of *h*_*c*_, *h*_*N*_, and Δ*h*.

In comparison with our inhomogeneous phase magnet model, we also examined a single-main-phase magnet model with a homogeneous composition equivalent to *δ* = 0.3 (Nd_1.51_Ce_0.49_Fe_14_B). The *h*_c_, *h*_*N*_, and Δ*h* values for the individual spheres were extracted using the aforementioned grain-by-grain analysis, as summarized in Fig. 9 and Supplementary Table S1 online. The mean values of *h*_c_ and *h*_*N*_ from this homogenous phase model were − 4.87 and − 4.86 T, respectively, excluding the four outlier values spanning over − 5.5 to − 5 T. These outlier values correspond to the delayed reversals of specific grains which are in small amount and have negligible effects on the overall coercive forces and nucleation fields. On the other hand, the standard deviations of *h*_c_ and *h*_*N*_ are 13.1 and 12.7 mT, respectively, which are less than those for the inhomogeneous phase model with *δ* = 0.3. The distribution of Δ*h* (mean value, 16.3 mT) for the single-main-phase magnet model is very similar to that for the inhomogeneous phase model. Hence, the calculated domain-wall speed was 1.88 km/s, roughly equivalent to those for MMP models. The standard deviation of Δ*h* was also less than that of MMP grains similar to the case of *h*_c_ and *h*_*N*_.

The overall nucleation field and coercive force, as explained in the previous sections, were superior in the single-main-phase magnet model compared to MMP models, which are composed of 28 Nd-rich grains and 27 Nd-lean grains. It is important to note that the Ce contents in the MMP or dual-main phase magnets, and single-main phase magnets are generally different from those in the starting materials due to the formation of precipitate phases around the thermodynamically stable RE_2_Fe_14_B-phase grains^28–30,32^.

## Summary

We utilized finite-element micromagnetic simulations to investigate the individual demagnetization curves of spheres with varying Nd-rich and -lean core-shells within a sphere- cluster model. The overall demagnetization curve from the entire inhomogeneous magnetic phases can be constructed as the summation of the demagnetization curves of the individual grains. Through our grain-by-grain analysis of the coercive force *h*_c_ and nucleation field *h*_*N*_ for each grain, we observed that the Nd-lean grains reversed (nucleated) at nearly identical (at least similar) values of *h*_*c*_ (*h*_*N*_). This was then followed by the intermittent reversal (nucleation) of the Nd-rich grains at widely varying values of *h*_*c*_ (*h*_*N*_). This can be attributed to the localized, irregular dipolar fields produced by the Nd-lean grains that had undergone reversal before the Nd-rich grains. Furthermore, our machine learning analysis using kernel SHAP interpretation indicates significant contributions from the stray fields of reversed Nd-lean grains to the reversals of Nd-rich grains in a wide switching field range. The similar narrow distributions of Δ*h* observed in both Nd-rich and -lean grains can be attributed to their comparable domain-wall mobilities.

Compared to the reversal of inhomogeneous magnetic phases (MMP), the single-main-phase model showed relatively narrow distributions ($\sigma_{{h_{c} }}^{SP} = 13.1{\text{ mT}}$, $\sigma_{{h_{N} }}^{SP} = 12.7{\text{ mT}}$) of *h*_*c*_ and *h*_*N*_, with mean values higher than those from the MMP model. We emphasize that the magnitude of *h*_*N*_ in the MMP model trended with the mean value of *h*_*N*_ for Nd-lean grains ($\left\langle {h_{N} } \right\rangle^{L}$) with respect to the *δ* values, while the magnitude of *h*_*c*_ with the mean value of *h*_c_ for Nd-rich grains ($\left\langle {h_{c} } \right\rangle^{R}$), owing to the distinct distributions of *h*_*N*_ and *h*_*c*_ values of the two types of grains. Furthermore, according to the proportionality between *h*_*c*_ (*h*_*N*_) and the anisotropy field (*h*_*A*_) in the Kronmüller relation, the mean values of *h*_*c*_ and *h*_*N*_ in Nd-lean grains increased with *δ* due to the addition of Nd atoms in their shell, while those in Nd-rich grains decreased due to Nd atoms being depleted from the shells.

This work offers guidance for the optimal design of granular hard magnets composed of Nd_2_Fe_14_B and other abundant rare earth transition elements, aiming for cost-effective performance through meticulous adjustment of microstructures and elemental compositions.

## Methods

### Micromagnetic simulations

The demagnetization curves of the sphere cluster model were numerically calculated using the finite-element micromagnetic simulation tool, FEMME^54^. This tool solves the Landau-Lifshitz-Gilbert equation^13^

$$\frac{{d{\mathbf{m}}}}{dt} = - \gamma \mu_{0} {\mathbf{m}} \times {\mathbf{H}}_{{{\text{eff}}}} + \alpha {\mathbf{m}} \times \frac{{d{\mathbf{m}}}}{dt}.$$

Here *γ* is the gyromagnetic ratio, *μ*_0_ the permeability of vacuum, *α* the damping constant, and **m** and **H**_eff_ the reduced magnetization (= *μ*_0_**M**/*J*_*S*_) and effective field. We used interpolated values from the material parameters of Nd_2_Fe_14_B (exchange constant *A*_*ex*_ = 7.7 pJ/m; saturation polarization *J*_*S*_ = 1.61 T; uniaxial anisotropy constant *K*_1_ = 4.3 MJ/m^3^) and Ce_2_Fe_14_B (exchange constant *A*_*ex*_ = 5.0 pJ/m; saturation polarization *J*_*S*_ = 1.17 T; uniaxial anisotropy constant *K*_1_ = 1.5 MJ/m^3^)^27^ to represent the given compositional alloy materials. The damping constant,* α*, was set to 1 to expedite convergence^14,55^. The initial magnetization configuration was assumed to be **m** = [0, 0, 1], and the external magnetic field was linearly decreased from *μ*_0_*H*_z_ =  + 7 T to − 7 T over a span of 70 ns.

Finite-element meshes on the sphere’s surface were constructed using geodesic polyhedrons. These are approximations of spheres composed of triangles (Fig. 1c), with edge lengths shorter than the exchange length (2.7 nm for Nd_2_Fe_14_B). This design ensures the correct evaluation of the exchange interaction term in the model system^1,14,54^. As the small sizes of micromagnetic cells allow for a precise description of domain walls (3–5 nm^56,57^), we restricted our mesh sizes in the finite-element simulations to be below $\min (l_{ex} ,l_{K} )$^54^, where $l_{ex} = \sqrt {{{2A_{ex} \mu_{0} } \mathord{\left/ {\vphantom {{2A_{ex} \mu_{0} } {J_{S}^{2} }}} \right. \kern-0pt} {J_{S}^{2} }}}$ and $l_{K} = \sqrt {{{A_{ex} } \mathord{\left/ {\vphantom {{A_{ex} } {K_{1} }}} \right. \kern-0pt} {K_{1} }}}$. In the present FEMME simulations, mesh sizes of ~ 2 nm were sufficient to accurately describe the domain wall configuration in the Nd_2_Fe_14_B material, with characteristic widths of $\delta_{DW}^{Bloch} = \pi l_{ex} = 4.24{\text{ nm}}$. The Class-I icosahedron-based geodesic polyhedrons, with a subdivision frequency of 19, have edge lengths of approximately 1.98 nm ({3,5 +}_19,0_ by Wenninger notation^58^). These were calculated using the open-source code, Antiprism^59^. Tetrahedron meshes were then generated inside the geodesic polyhedrons using the TetGen software^60^.

### Optimization of machine learning models

To develop the machine learning models, we generated 100 artificial neural network (ANN) models using the scikit-learn package in Python. We employed the MLPRegressor function from the scikit-learn package, which generates a single-layered artificial neural network trained via backpropagation. The hidden layer consisted of 100 nodes, while the input layer took nucleation fields of the core and shell parts from 55 grains (110 in total). These were divided into a training set of 77 and a test set of 33. Due to the small size of the datasets, we trained 100 machine learning models with various hyperparameters determined by the Very Fast Simulated Annealing (VFSA) algorithm^24^. Nevertheless, the majority of our machine learning models showed fairly good predictions, with *R*^2^ > 0.92. Our VFSA algorithm incorporated an adaptive cooling schedule,

$$T_{j + 1} = \frac{{T_{j} }}{{1 + \exp \left[ { - \left( {f({\mathbf{x}}_{cand} ) - f({\mathbf{x}}_{curr} )} \right)/T_{0} } \right]}}$$

where $f({\mathbf{x}})$, ${\mathbf{x}}_{cand}$, and ${\mathbf{x}}_{curr}$ indicate the objective function, candidate solution, and current solution, respectively. The candidate solution for hyperparameters was searched for 30 times at each temperature, continuing until the current temperature reached 10^–30^.

### Kernel SHAP interpretation

The trained machine learning models were analyzed with kernel SHAP interpretation implemented, as implemented by the alibi package^34^. According to Ref.^34^, the importance values of machine learning models are calculated from the contribution of the *i*-th feature out of *M* features. The output of the model, denoted as *f*, with reference to the average output *f*_0_ is

$$f({\mathbf{x}}) = g({\mathbf{x}}^{\prime } ) = f_{0} + \sum\limits_{i = 1}^{M} {x_{i}^{\prime } \phi_{i} } ,$$

where $g({\mathbf{x}}^{\prime } )$ and ${\mathbf{x^{\prime}}} \in \{ 0,1\}^{M}$ are the explainable model and coalition vector, respectively.

### Supplementary Information

Supplementary Information 1.

Supplementary Video 1.

## Data Availability

The data that support the findings of this study are available from the corresponding author upon reasonable request.

## References

[1] Fidler J, Donahue M (2020). Hard magnets. Compendium on Electromagnetic Analysis: From Electrostatics to Photonics: Fundamentals and Applications for Physicists and Engineers Volume 1 Electrostatic and Magnetic Phenomena.

[2] Coey JMD (2020). Perspective and prospects for rare earth permanent magnets. Engineering.

[3] Jiles D (2015). Introduction to Magnetism and Magnetic Materials.

[4] Stoner EC, Wohlfarth EP (1948). A mechanism of magnetic hysteresis in heterogeneous alloys. Philos. Trans. R. Soc. A.

[5] Ntallis N (2022). Macrospin model of an assembly of magnetically coupled core-shell nanoparticles. Phys. Rev. B.

[6] El-Hilo M, de Witte AM, O’Grady K, Chantrell RW (1992). The sweep rate dependence of coercivity in recording media. J. Magn. Magn. Mater..

[7] Feng X, Visschera PB (2004). Sweep-rate-dependent coercivity simulation of FePt particle arrays. J. Appl. Phys..

[8] Plumer ML, Leblanc MD, Whitehead JP, van Ek J (2012). Micromagnetic simulations of sweep-rate dependent coercivity in perpendicular recording media. J. Appl. Phys..

[9] Carrey J, Mehdaoui B, Respaud M (2011). Simple models for dynamic hysteresis loop calculations of magnetic single-domain nanoparticles: Application to magnetic hyperthermia optimization. J. Appl. Phys..

[10] Raquet B, Mamy R, Ousset JC (1996). Magnetization reversal dynamics in ultrathin magnetic layers. Phys. Rev. B.

[11] Zirka SE, Moroz YI, Harrison RG, Chwastek K (2012). On physical aspects of the Jiles-Atherton hysteresis models. J. Appl. Phys..

[12] Van de Wiele B, Vandenbossche L, Dupré L, De Zutter D (2010). Energy considerations in a micromagnetic hysteresis model and the Preisach model. J. Appl. Phys..

[13] Gilbert TL (2004). A phenomenological theory of damping in ferromagnetic materials. IEEE Trans. Magn..

[14] Kronmüller H, Fähnle M (2003). Micromagnetism and the Microstructure of Ferromagnetic Solids.

[15] Kovacs A (2020). Computational design of rare-earth reduced permanent magnets. Engineering.

[16] Dengina E, Bolyachkin A, Sepehri-Amin H, Hono K (2022). Machine learning approach for evaluation of nanodefects and magnetic anisotropy in FePt granular films. Scr. Mat..

[17] Krone P, Makarov D, Albrecht M, Schrefl T, Suess D (2010). Magnetization reversal processes of single nanomagnets and their energy barrier. J. Magn. Magn. Mater..

[18] Toson P, Asali A, Wallisch W, Zickler G, Fidler J (2015). Nanostructured hard magnets: A micromagnetic study. IEEE Trans. Magn..

[19] Niarchos D (2015). Toward rare-earth-free permanent magnets: A combinatorial approach exploiting the possibilities of modelling, shape anisotropy in elongated nanoparticles, and combinatorial thin-film approach. JOM.

[20] Exl L (2019). Magnetic microstructure machine learning analysis. J. Phys. Mater..

[21] Kim S-K, Hwang S, Lee J-H (2019). Effect of misalignments of individual grains’ easy axis on magnetization reversal process in granular NdFeB magnets: A finite-element micromagnetic simulation study. J. Magn. Magn. Mater..

[22] Gusenbauer M (2020). Extracting local nucleation fields in permanent magnets using machine learning. npj Comput. Mater..

[23] Tsukahara H, Iwano K, Ishikawa T, Mitsumata C, Ono K (2020). Relationship between magnetic nucleation and the microstructure of a hot-deformed permanent magnet: micromagnetic simulation. NPG Asia Mater..

[24] Park H-K, Lee J-H, Lee J, Kim S-K (2021). Optimizing machine learning models for granular NdFeB magnets by very fast simulated annealing. Sci. Rep..

[25] Bao L, Yun G, Bai N, Cao Y (2021). Grain-size effect on coercivity of Nd–Fe–B nanomagnets: micromagnetics simulation based on a multi-grain model. Appl. Phys. Express.

[26] Behbahani R, Plumer ML, Saika-Voivod I (2022). Micromagnetic simulations of clusters of nanoparticles with internal structure: Application to magnetic hyperthermia. Phys. Rev. Appl..

[27] Liu D (2017). Micromagnetic simulation of the influence of grain boundary on cerium substituted Nd-Fe-B magnets. AIP Adv..

[28] Sasaki TT (2016). Formation of non-ferromagnetic grain boundary phase in a Ga-doped Nd-rich Nd–Fe–B sintered magnet. Scr. Mater..

[29] Soderžnik M (2017). Magnetization reversal of exchange-coupled and exchange-decoupled Nd-Fe-B magnets observed by magneto-optical Kerr effect microscopy. Acta Mater..

[30] Ma T (2018). Grain boundary restructuring of multi-main-phase Nd–Ce–Fe–B sintered magnets with Nd hydrides. Acta Mater..

[31] Lee J-H, Choe J, Hwang S, Kim S-K (2017). Magnetization reversal mechanism and coercivity enhancement in three-dimensional granular Nd–Fe–B magnets studied by micromagnetic simulations. J. Appl. Phys..

[32] Jin J, Ma T, Zhang Y, Bai G, Yan M (2016). Chemically inhomogeneous RE-Fe-B permanent magnets with high figure of merit: Solution to global rare earth criticality. Sci. Rep..

[33] Kim C (2021). Micromagnetic simulation of microstructure effect for binary-main-phase Nd–Ce–Fe–B magnets. J. Phys. D.

[34] Lundberg, S. M. & Lee, S.-I. A unified approach to interpreting model predictions. 10.48550/arXiv.1705.07874 (2017).

[35] Ferrando R (2016). Structure and Properties of Nanoalloys.

[36] Bleaney B, Hull RA (1941). The effective susceptibility of a paramagnetic powder. Proc. R. Soc. Lond. Ser. A.

[37] Bjørk R, Bahl CRH (2013). Demagnetization factor for a powder of randomly packed spherical particles. Appl. Phys. Lett..

[38] Normile PS (2016). Demagnetization effects in dense nanoparticle assemblies. Appl. Phys. Lett..

[39] Zhao GP, Wang XL, Yang C, Xie LH, Zhou G (2007). Self-pinning: Dominant coercivity mechanism in exchange-coupled permanent/composite magnets. J. Appl. Phys..

[40] Zhao GP, Wang XL (2006). Nucleation, pinning, and coercivity in magnetic nanosystems: An analytical micromagnetic approach. Phys. Rev. B.

[41] Mougin A (2007). Domain wall mobility, stability and Walker breakdown in magnetic nanowires. EPL.

[42] Fernandez-Roldan JA (2019). Modeling magnetic-feld-induced domain wall propagation in modulated-diameter cylindrical nanowires. Sci. Rep..

[43] Usov NA, Nesmeyanov MS (2020). Multi-domain structures in spheroidal Co nanoparticles. Sci. Rep..

[44] Moreno R, Carvalho-Santos VL, Altbir D, Chubykalo-Fesenko O (2022). Detailed examination of domain wall types, their widths and critical diameters in cylindrical magnetic nanowires. J. Magn. Magn. Mater..

[45] Jackson JD (1962). Classical Electrodynamics.

[46] Bjørk R, Insinga AR (2023). Explaining Browns paradox in NdFeB magnets from micromagnetic simulations. J. Magn. Magn. Mater..

[47] Brown WF (1963). Micromagnetics.

[48] Dobrynin AN, Barthem VMTS, Givord D (2009). Revisiting magnetization processes in granular hard magnetic materials. Appl. Phys. Lett..

[49] Bance S (2014). Grain-size dependent demagnetizing factors in permanent magnets. J. Appl. Phys..

[50] Givord D, Dobrynin AN (2019). Demagnetising fields in assemblies of magnetostatically coupled Stoner-Wohlfarth particles. J. Magn. Magn. Mater..

[51] Ishii Y, Nakazawa Y (1997). Magnetization curling in a disk with a uniaxial anisotropy. J. Appl. Phys..

[52] Ramesh R, Srikrishna K (1988). Magnetization reversal in nucleation controlled magnets. I.. Theory. J. Appl. Phys..

[53] Fischbacher J (2018). Searching the weakest link: Demagnetizing fields and magnetization reversal in permanent magnets. Scr. Mater..

[54] Suess D (2002). Time resolved micromagnetics using a preconditioned time integration method. J. Magn. Magn. Mater..

[55] Kikuchi R (1956). On the minimum of magnetization reversal time. J. Appl. Phys..

[56] Zhao GP, Zhao L, Shen LC, Zou J, Qiu L (2019). Coercivity mechanisms in nanostructured permanent magnets. Chin. Phys. B.

[57] Si W (2015). Deterioration of the coercivity due to the diffusion induced interface layer in hard/soft multilayers. Sci. Rep..

[58] Wenninger MJ (1971). Polyhedron Models.

[59] *Antiprism - Polyhedron Modelling Software*. https://www.antiprism.com (2019).

[60] Si H (2015). TetGen a delaunay-based quality tetrahedral mesh generator. ACM Trans. Math. Softw..

