# Supplementary material for: Magnetization reversals in core–shell sphere clusters: finite-element micromagnetic simulation and machine learning analysis
Source: Sci Rep. 2023 Sep 14;13:15240. doi: 10.1038/s41598-023-42498-z (PMC10502040; doi:10.1038/s41598-023-42498-z)
Supplement: Supplementary file 1 — Supplementary Information 1. [file 41598_2023_42498_MOESM1_ESM.pdf]

Supplementary Information for

Grain-by-grain analysis of magnetization reversals in a sphere-cluster model with core-shell inhomogeneous magnetic phases: A study of finite-element micromagnetic simulation and machine learning

Hyeon-Kyu Park and Sang-Koog Kim[a]

National Creative Research Initiative Center for Spin Dynamics and Spin-Wave Devices, Nanospinics Laboratory, Research Institute of Advanced Materials, Department of Materials Science and Engineering, Seoul National University, Seoul 151-744, South Korea

[a)] Authors to whom correspondence should be addressed. Electronic mail: sangkoog@snu.ac.kr

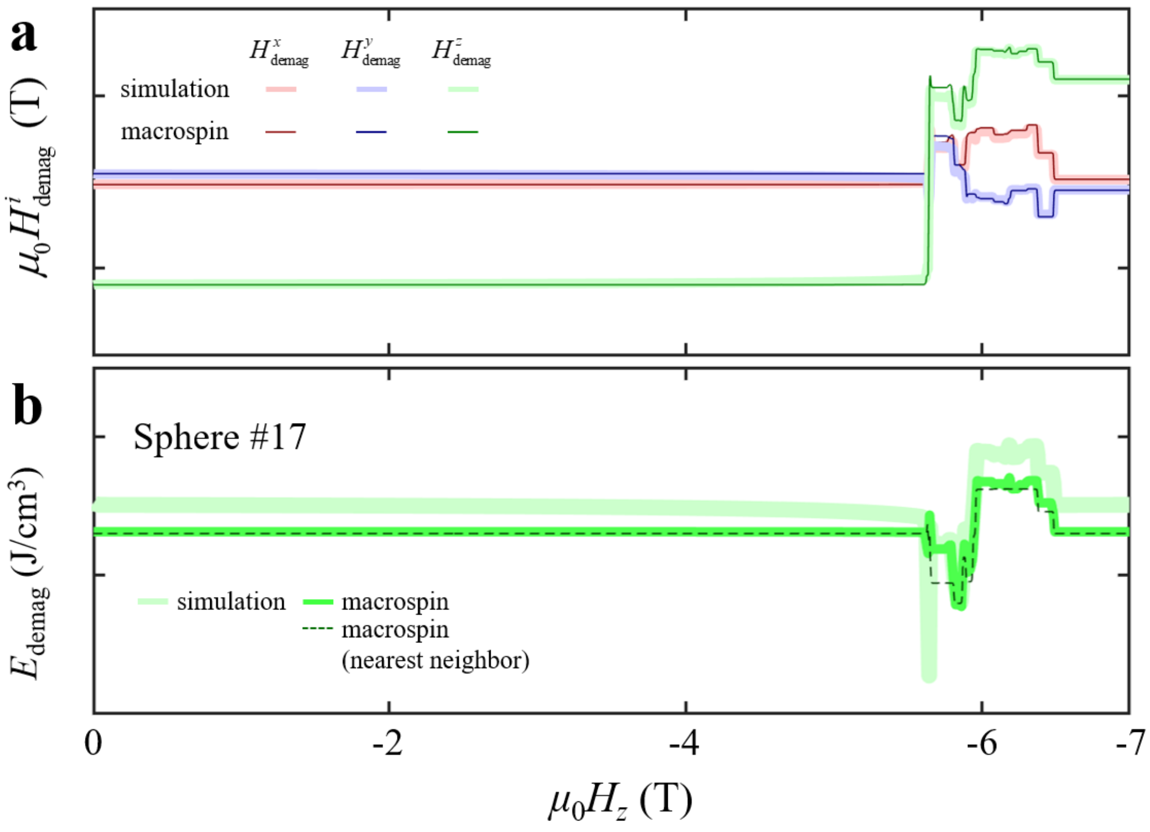

Figure S1. Validation of the analytical stray field formula based on the macrospin model (Eq. (3) in the main text). (a) The volume-average analytical stray field and the demagnetizing field from micromagnetic simulations for sphere #17 are plotted component-wise in different colors: the *x*-, *y*-, and *z*-component by red, blue, and green, respectively. (b) The curves of demagnetization energy density, calculated from the stray field formula (solid green line), the stray field formula considering only the nearest neighbor (dotted green line), and the micromagnetic simulation (light-green line), are plotted a function of the external magnetic field.

Table S1. Mean values and standard deviations of h_c, h_N, and Δh distributions for inhomogeneous magnetic phase (MMP) model versus homogeneouos single phase model, with $\delta = 0.3$.

Metrics		$\langle h_c \rangle$ (T)	σ_{h_c} (mT)	$\langle h_N \rangle$ (T)	σ_{h_N} (mT)	$\langle \Delta h \rangle$ (mT)	$\sigma_{\Delta h}$ (mT)
MMP	Nd-rich	-5.07	416.7	-5.06	418.0	19.5	3.20
	Nd-lean	-4.29	18.5	-4.28	17.8	17.0	2.76
single phase		-4.87	13.1	-4.86	12.7	16.3	2.38

Table S2. Mean values and standard deviations of h_c, h_N, and Δh distributions for inhomogeneous magnetic phase model with varying values of δ .

Grain type	δ	$\langle h_c \rangle$ (T)	σ_{h_c} (mT)	$\langle h_N \rangle$ (T)	σ_{h_N} (mT)	$\langle \Delta h \rangle$ (mT)	$\sigma_{\Delta h}$ (mT)
Nd-rich	0	-5.15	396.6	-5.14	397.5	18.6	3.44
	0.1	-5.12	389.6	-5.11	390.7	19.0	3.49
	0.2	-5.08	394.8	-5.07	395.9	18.9	3.67
	0.3	-5.07	416.7	-5.06	418.0	19.5	3.20
	0.4	-5.03	418.0	-5.02	419.3	19.7	3.76
	0.5	-4.98	420.6	-4.97	422.0	20.4	3.71
Nd-lean	0	-4.13	19.0	-4.12	18.6	19.1	3.64
	0.1	-4.17	20.5	-4.16	19.9	18.5	3.48
	0.2	-4.23	19.3	-4.22	18.7	17.7	2.81
	0.3	-4.29	18.5	-4.28	17.8	17.0	2.76
	0.4	-4.31	18.6	-4.31	17.8	16.6	2.12
	0.5	-4.34	17.6	-4.33	16.9	16.0	2.34

Movie S1. Magnetization reversal process of sphere cluster model. The Nd-lean grains are first switched in a narrow range (~0.1 T) of magnetic fields (blue curve in the right), followed by the reversal of Nd-rich grains in a much wider range (~1.5 T) of magnetic fields (red curve).
